# Coupling of Algal Biofuel Production with Wastewater

**DOI:** 10.1155/2014/210504

**Published:** 2014-05-26

**Authors:** Neha Chamoli Bhatt, Amit Panwar, Tara Singh Bisht, Sushma Tamta

**Affiliations:** Algae Laboratory, Department of Biotechnology, Kumaun University, Bhimtal Campus, Bhimtal, Nainital, Uttarakhand-263136, India

## Abstract

Microalgae have gained enormous consideration from scientific community worldwide emerging as a viable feedstock for a renewable energy source virtually being carbon neutral, high lipid content, and comparatively more advantageous to other sources of biofuels. Although microalgae are seen as a valuable source in majority part of the world for production of biofuels and bioproducts, still they are unable to accomplish sustainable large-scale algal biofuel production. Wastewater has organic and inorganic supplements required for algal growth. The coupling of microalgae with wastewater is an effective way of waste remediation and a cost-effective microalgal biofuel production. In this review article, we will primarily discuss the possibilities and current scenario regarding coupling of microalgal cultivation with biofuel production emphasizing recent progress in this area.

## 1. Introduction

World biofuel production has increased sevenfold since 2000 but still meets only 2.3% of final liquid fuel demands [1]. Worldwide energy consumption is projected to increase by 49% from 522 exajoules (EJ) in 2007 to 780 EJ in 2035 [2]. It is expected that two main energy resources, crude oil and natural gas, will be diminished by 45.7 and 62.8 years and estimated energy requirement will be tripled in 2025 [3]. Transportation is one of the fastest growing sectors using 27% of primary energy in current scenario and in India annual oil consumption is about 5.5% which is going to increase in the next decade [4]. Currently in India, diesel alone meets an estimated 73% of transportation fuel demand followed by gasoline at 20%; moreover, it is estimated that, by the end of this decade, the average demand for transport fuels will rise from an estimated 117 billion litres in 2013 to 167 billion litres and would grow further to reach 195 million liters by 2023 [5]. Fossil fuels make up 88% and 86.2% of the energy consumed by the world and USA [6]. The transportation and energy sectors are the major sources in European Union (EU) responsible for more than 20% and 60% of greenhouse gas (GHG) emissions, respectively [7]. It is fully accepted that global warming increases in response to (GHG) emissions [8] which reveals that there is vital need for cleaner alternative to fossil fuels. Due to continuous depletion of natural resources emerging energy crisis and increasing fuel prices demands for an alternative, scalable and sustainable energy source. Moreover, the entire globe is facing two major challenges of freshwater shortage and energy crisis [9]. Recently, renewable energies including water, wind, solar, geothermal and especially biofuels have gained much attention as a substitute to conventional energy resources as they are sustainable, ecofriendly, and carbon neutral and have potential to fulfil the energy needs of the transportation sector.

Microalgae have potential to become an alternative source of petrodiesel due to their sustainable photosynthetic efficiency, ecofriendly approach, increasing depletion of nonrenewable energy resources, and use of nonarable land. Coupling wastewater with microalgae cultivation can be a promising approach for biofuel production. This integration can offer an economically viable and environmentally friendly means for sustainable algal biofuel production since enormous amounts of water and nutrient (e.g., nitrogen and phosphorus) can be recycled for algal growth in wastewater-based algal cultivation system [10, 11]. Microalgae have dual application of biomass production for sustainable biofuels production and phycoremediation [12]. They have higher photosynthetic efficiency and lipid content which can be harnessed for biofuels including biodiesel, bioethanol, biohydrogen, and combustible gases. Microalgal biofuel systems are capable of producing clean and sustainably produced fuels for the future while eradicating the food versus fuel and forest versus fuel concerns associated with first generation biofuels and lignocellulosic processes based on wood feedstocks [13]. Although there had been enough debate on algal biofuels, still they are not commercialized as their economic viability is questionable. Despite being so advantageous, the technoeconomics of present microalgal biofuel production systems is not effective to compete with petroleum-based conventional fuels as it is accompanied with high cost production. The major expenditure of a current algae biofuel technology depends on algae cultivation system, harvesting, and lipid extraction methods. But certainly their outlook is promising, and both the developed nations and the emerging economies are interested in algal fuels [14].

## 2. Microalgae: Advantages as a Source of Biofuel

Microalgae are prokaryotic or eukaryotic photosynthetic microorganisms, some of which can also form a chain or colony ranging from a few micrometers to a few hundred micrometers and are generally ubiquitous in nature. Algae are a broad category that has no proper taxonomic classification [15]. They are primitive plants (thallophytes), that is, lacking roots, stems, and leaves, have no sterile covering of cells around the cells, and have chlorophyll as their primary photosynthetic pigment [16]. Algae can be divided into two main categories, that is, macroalgae which are multicellular and size up to several meters and microalgae are small size organisms ranging from sizes 0.2 *μ*m to 100 *μ*m or even higher [17]. Microalgae have high rate of areal biomass productivity compared to traditional agricultural crops like corn and soya bean and oil content in microalgae can exceed 80% dry weight of biomass [18]. Generally algae are divided into five main groups:

- blue-green algae (Cyanophyceae),
- green algae (Chlorophyceae),
- diatoms (Bacillariophyceae),
- red algae (Rhodophyceae),
- brown algae (Phaeophyceae).

Although Cyanobacteria (blue-green algae) are classified to the domain of bacteria, being photosynthetic prokaryotes, they are often considered as “algae” [19]. There are many promising attributes of microalgae which make them desirable for biofuel production. The main source of carbon for the growth of microalgae is atmospheric carbon dioxide [20]. Several microalgae species can grow well on nonpotable water (brackish, wastewater, and seawater); their is a possibility that biofuel production can be coupled with one of these systems in future. This coupling does not compete for arable land which can be used for agricultural purpose and also for eliminating the use of freshwater resources. The production of biofuels from algae can be coupled with flue gas CO_2_ mitigation, wastewater treatment, and production of high-value chemicals [21, 22]. Many species of microalgae produce significant quantities of lipids which can be converted to biodiesel through process of transesterification. Microalgal biodiesel has characteristics related to petroleum-based diesel including density, viscosity, flash point, cold flow, and calorific value. Microalgae can be harvested batch-wise nearly all year round providing a reliable and constant supply of oil [20]. Microalgae does not require the use of chemical unlike terrestrial plants requiring herbicides or pesticides which affect the environment adversely and increase the cost of production. Lignin is generally absent in microalgae and other large biopolymers (found in woody biomass) that may hinder with biomass processing and conversion [23]. Apart from this, the residual algal biomass mainly composed of proteins and carbohydrates can be processed to various biofuels, including methane and alcohol fuels, and it can also produce other nonfuel coproducts which can be recovered and formulated into products with high market value such as nutriceuticals, therapeutics, and animal feeds [24].

## 3. Microalgal Farming Using Wastewater for Biofuel Production

Due to global expansion of human population and advanced living standards of people, a high level of water pollution is generated worldwide. Wastewater is basically end product generated by domestic, municipal, agricultural, and industrial sources [25]. The composition of wastewater is a reflection of the life styles and technologies practiced in the producing society [26]. Wastewater generally contains organic mass like proteins, carbohydrates, lipids, volatile acids, and inorganic content containing sodium, calcium, potassium, magnesium, chlorine, sulphur, phosphate, bicarbonate, ammonium salts, and heavy metals [27]. Excess of these nutrient loads in surrounding water bodies causes eutrophication or algal blooms often due to anthropogenic waste production.

More than 300 million tonnes of biodegradable household and household-like wastes, industrial wastes, and other wastes are generated every year in the European Union and stay mostly unexploited [45]. Human beings generate approximately ~3 billion tonnes of domestic wastewater every year [46]. In India, annually, due to migration of people into cities, the figures are expected to reach about 600 million by 2030 making and simultaneously increasing pressure on urban return flow (wastewater) which is usually about 70–80% of the water supply [47]. The recent reports of Central Pollution Control Board [48], New Delhi, India, revealed that the wastewater generation in the nation is around 40 billion litres per day largely from urban areas and ironically only 20–30% of the generated wastewater is subjected to treatment. In majority of the developing nations, the main sources of wastewater generation are domestic, municipal, agricultural, and industrial activities which are foremost released into environment without having sufficient treatment steps. Many species of microalgae are able to efficiently grow in wastewater environment through their capability to use abundant organic carbon and inorganic N and P in the wastewater [11]. Algae take up these nutrients along with CO_2_ and produce biomass through the process of photosynthesis. Microalgae are the main microorganisms used in treatment of domestic wastewater in units such as oxidation ponds or oxidation ditches. Algae have also been deployed for low cost and environmentally friendly wastewater treatment [49–51]. The idea of coupling of wastewater as a medium for biofuel production from algae is not innovative, as it was previously suggested in report of the Aquatic Species Program (ASP) conducted from 1978 to 1996, U.S.A [10]. The main constraint for wastewater-based algae biofuel production system is to find ideal microalgae strains which could be able to grow in wastewater environment with significant nutrient removal efficiency showing high biomass and lipid productivity.

Extensive work has been conducted by researchers [35, 37, 40, 52–54] from numerous parts of the world to explore the feasibility of using microalgae for biofuel potential from wastewater with nutrient removal property particularly nitrogen and phosphorus from effluents. Researchers had more focus on microalgal culture for N and P removal from domestic sewage in comparison to industrial wastewater. The reason behind this is that industrial wastewater, such as tannery wastewater and chemical industry wastewater, has more metal ions in addition to various organic N and P compounds [39] and it is more toxic having heavy metal contamination which does not facilitate the algal growth. The total biofuel potential of algae, when grown in domestic wastewater generated by 1000 urban centres in India, is ~0.16 Mt/annum considering the lipid fraction as 20% [55].

### 3.1. Biomass Production from Wastewater Grown Microalgae

Species selection, optimization of growth, lipid content, and harvesting at large scale are the important factors which govern the commercialization potential of algal biofuels. The algal biomass produced and harvested from these wastewater treatment systems could be transformed through a variety of pathways to biofuels, for example, anaerobic digestion to biogas, transesterification of lipids to biodiesel, fermentation of carbohydrate to bioethanol, and high temperature alteration to biocrude oil [56]. Economic feasibility of algal biofuel production from wastewater treatment can be achieved by high rate algal ponds (HRAPs) with low environmental impact compared to commercial algal production by HRAPs which consume freshwater and fertilisers [57]. The major challenge in the microalgae research for the existing high rate ponds is designing an efficient and economical carbonation system to fulfill the needs of high CO_2_ requirement that might improve the biomass productivity [58]. Viswanath and Bux [59] isolated* Chlorella *sp. from wastewater pond and screened it for its efficiency in lipid production by cultivating both photoautotrophic and heterotrophic conditions in bioreactor. Maximum amount of biomass was recovered from the* Chlorella *sp. grown under heterotrophic growth conditions with 8.90 gL^−1^ compared to photoautotrophic growth conditions which were about 3.6-fold lesser than the former, resulting in the accumulation of high lipid content in cells compared to autotrophic growth by enhancing lipid production by 4.4-fold. This study suggested that heterotrophic growth of microalgae is an efficient method for the production of biomass and high lipid content in the cells, which can reduce the cost of microalgal biomass production.

## 4. Harvesting Methods of Microalgae

**Figure 1:**
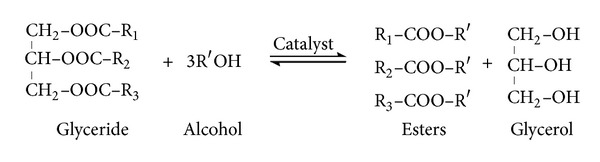
The chemical reaction of transesterification process.

Dewatering and harvesting of algal biomass are a necessary step for concentrating algal biomass and for further releasing triacylglycerols (TAGs) which can then be transesterified to produce biodiesel (as shown in Figure 1). Harvesting and dewatering are one of the challenging areas of current biofuel technology as microalgae have small size and low density increasing the capital cost. The difficulty is in releasing the lipids from their intracellular location in the most energy-efficient and economical way possible, avoiding the use of large amounts of solvent, such as hexane, and utilising as much of the carbon in the biomass as liquid biofuel as possible, potentially with the recovery of minor high-value products [60]. The major techniques presently applied in microalgae harvesting and recovery include centrifugation, flocculation, filtration, and flotation.

### 4.1. Flocculation

Flocculation is a process of forming aggregates known as algae flocs that are often performed as a pretreatment to destabilize algae cells from water and to increase the cell density by natural, chemical, or physical means. Chemicals called flocculants are usually added to induce flocculation, and commonly used flocculants are inorganic flocculants such as alum [61, 62] or organic flocculants such as chitosan [63] or starch [64]. The surface charge of microalgal cells is generally negatively charged due to the ionization of functional groups on the microalgal cell walls and also by the adsorption of ions from the culture medium which can be neutralized by applying positively charged electrodes and cationic polymers, also commonly used to flocculate the microalgal biomass. This harvesting method is pretty expensive because of the cost of flocculants; hence flocculants need to be inexpensive, easily produced, and nontoxic.

### 4.2. Centrifugation

Centrifugation is a widely used method of separation on the basis of particle size and density separation. Separation efficiency is dependent upon the size of desired algal species. Numerous centrifugal techniques have been employed in various types and sizes depending on the uses such as tubular centrifuge, multichamber centrifuges, imperforate basket centrifuge, decanter, solid retaining disc centrifuge, nozzle type centrifuge, solid ejecting type disc centrifuge, and hydrocyclone [65]. Despite being energy intensive method, it is rapid and a preferred method for microalgal cell recovery, whereas cell viability was found to be significantly dependent on the microalgal species and the method of centrifugation [66]. Even though it is very effective, centrifugation is considered unfeasible in large-scale algal culture system due to the high capital and operational costs.

### 4.3. Filtration

Filtration harvests microalgal biomass through filters on which the algae accumulate forming thick algae paste and allow the liquid medium to pass through. Filtration systems can be classified as macrofiltration (pore size of >10 *μ*m), microfiltration (pore size of 0.1–10 *μ*m), ultrafiltration (pore size of 0.02–2 *μ*m), and reverse osmosis (pore size of <0.001 *μ*m) [67]. There are many different forms of filtration, such as dead end filtration, microfiltration, ultrafiltration, pressure filtration, vacuum filtration, and tangential flow filtration (TFF) [68]. Nevertheless, it is accompanied with extensive running costs and time consuming.

### 4.4. Flotation

Microalgae cells are trapped on microair bubbles and float at the surface of water [69]. Generally, the flotation efficiency is dependent on the size of the created bubble: nanobubbles (<1 *μ*m), microbubbles (1–999 *μ*m), and fine bubbles (1–2 mm) [70]. Dissolved air flotation is a widely used technique in which microalgal cells are usually flocculated first and then air is bubbled through the liquid causing the flocs to float to the surface for easier harvesting. Hydrophobic interaction and surface charge of microalgae play crucial role for attachment of microalgae to the bubbles.

## 5. Lipid Extraction Methods

Lipid is polymer of fatty acids which is generally hydrophobic in nature and is classified as a polar (membrane lipids) and nonpolar lipid (neutral lipids). Polar lipids interact with polar solvents like ethanol and methanol and similarly nonpolar lipids interact with nonpolar solvents like chloroform and benzene which is the basis of designing solvent system for lipid extraction methods [71]. This solvent system mainly disrupts noncovalent interactions, hydrophobic interactions, and hydrogen bonding between lipid and associated macromolecule like protein. Lipids are mainly composed of 90–98% (weight) of TAGs, small amounts of mono- and diglycerides, free fatty acids (1–5%), and residual amounts of phospholipids, phosphatides, carotenes, tocopherols, sulphur compounds, and traces of water [72]. The majority of the lipid fraction is comprised of TAG content, an important parameter for biodiesel production. The saturated (16 : 0) and monounsaturated (18 : 1) fatty acids also play essential role in determining the fuel properties of biodiesel such as cetane number, oxidative stability, and cold flow [73]. Usually, extraction of lipid from microalgae consists of dual steps: cell disruption and solvent extraction. The lipid extraction methods might work differently on a variety of algal species as algae have an enormous variation in cell shape, size, cell wall composition, and types of algal lipids.

Microalgae cell disruption can be achieved by sonication, homogenisation, grinding, bead beating, or freezing [74]. The Folch et al. [75] method was originally optimized for isolation and purification of total lipids from animal tissues is also used for the extraction of total lipids from microalgae using chloroform-methanol 2 : 1. The most commonly used method for total lipid extraction from microalgae is the Bligh and Dyer method [76] which uses chloroform and methanol in 1 : 2. Another method for extraction of biooil or other molecule is to carry out extraction using safe and nonflammable solvent supercritical carbon dioxide (SC-CO_2_) which solubilizes non polar compounds; when the molecule of interest is not soluble, the solvent power can be increased using a safe and polar modifier, such as ethanol [77]. But it has few disadvantages; it is uneconomical for large-scale, energy-consuming step of drying pretreatment which limits its application for biofuel production. Ryckebosch et al. [78] optimized procedure for extraction of total and nonpolar lipids from microalgae showing chloroform-methanol 1 : 1 to be the best solvent mixture for extraction of total lipids from microalgae. Sathish and Sims [79] developed wet lipid extraction procedure capable of extracting 79% of transesterifiable lipids from wet algal biomass (84% moisture) via acid and base hydrolysis (90°C and ambient pressures), and 76% of extracted lipids were further converted to FAMEs. Ultimately, it is necessary to develop extraction method having less organic solvent use, reduce contamination, and avoid drying of algae to obtain significant cost reduction for scaling up mass algal culture system for biodiesel production.

### 5.1. Lipid Analysis Methods

After lipid extraction, it is important to identify and quantify lipid contents to screen the desirable algal strains for their biofuel efficiency. Further quantification of lipids requires separation of the crude extract and quantification of the lipid fraction by thin-layer chromatography (TLC), HPLC, or gas chromatography (GC) [80]. The Nile red fluorescence method is also employed for the determination of both neutral and polar lipids in algae but has been unsuccessful in many others, particularly in those with thick rigid cell walls that prevent the penetration of this lipid soluble fluorescence dye [81]. Mostly, microalgal lipid profiling is done by gas chromatography with flame ionization detector (GC-FID) and is carried out using the methylated ester form of the lipid [82].

### 5.2. Transesterification

Transesterification or alcoholysis is the reaction of a lipid with an alcohol to form esters and a by-product, glycerol [71]. This reaction actually converts highly viscous raw lipid/oil into low molecular weight molecules in the form of fatty acid alkyl esters which can be used as an alternative fuel for diesel engines. Biodiesel is a term used to describe “fuel comprised of monoalkyl esters of long-chain fatty acids that are derived from vegetable oils or animal fats” [83]. In a typical biodiesel reaction, TAGs enter into a reaction with methanol which yields fatty acid methyl esters (biodiesel) and glycerol as a waste product (as shown in Figure 1). Mainly three approaches are used to produce biodiesel; they are base catalyzed transesterification, acid catalyzed transesterification (with simultaneous esterification of free fatty acids), and noncatalytic conversion [84, 85].

## 6. Lipid Productivity from Wastewater Grown Algae

Lipid productivity takes into account both the lipid concentration within cells and the biomass produced by these cells and is therefore a more useful indicator of the potential costs of liquid biofuel production [86]. High lipid productivity is a key characteristic of a microalgal species for biodiesel production [87]. Generally, algae produce lipids between C_14_ and C_20_ in length. During stressful conditions like nutrient limitation, algae change their biosynthetic pathways and produce TAGs, which accumulate in the cytoplasm for the purpose of energy and carbon storage [88, 89]. Nevertheless, deliberately, cultivation of algae in stressful conditions can inhibit cell division, leading to decrease in overall lipid productivity [33, 90]. This is one of the reasons that it is difficult to maximize both high biomass and lipid productivity simultaneously. But still to achieve substantial cost reduction for the commercialization of biofuel production; it is suggested that the near-term research should be focused on maximizing lipid content with the help of altering the phycological metabolism of the microalgal cell and manipulation of cultivation system with the application of advanced engineering and design system.

Although, in many cases, most of the microalgal species reported with high lipid content does not adapt well to grow in wastewater, many researchers had screened microalgal isolates able to grow in wastewater effluents showing high lipid content. Xin et al. [54] isolated a freshwater microalga* Scenedesmus *sp. LX1 with high lipid content (around 25–35%) from low nutrient environment, and they compared* Scenedesmus *sp. LX1 with other reported 11 oily microalgal species based on the growth and lipid accumulation properties while growing in the secondary effluent of domestic wastewater.* Scenedesmus *sp. LX1 showed best growth and accumulated the maximum lipid content in microalgal cells in comparison to other microalgal species which could not grow in the secondary effluent of domestic wastewater. Zhou et al. [35] screened 17 top-performing strains isolated from water bodies including wastewater from Minnesota which grew well in centrate (highly concentrated municipal) wastewater. Five strains were promising, that is,* Chlorella *sp.*, Heynigia *sp.,* Hindakia *sp.,* Micractinium *sp., and* Scenedesmus *sp., regarding their ability to adapt to centrate municipal wastewater showing high growth rates (0.455–0.498 d^−1^) and higher lipid productivities (74.5–77.8 mg L^−1^ d^−1^). Bhatnagar et al. [52] isolated three robust mixotrophic microalgae isolated from industrial wastewater and evaluated their growth potential in media supplemented with different organic carbon substrates and wastewaters, which showed 3–10 times more biomass production relative to phototrophy. Devi et al. [53] evaluated the effect of sequential growth phase (GP) and starvation phase (SP) on the lipid productivity of heterotrophically grown mixed microalgae using domestic wastewater as a substrate. The mixotrophic microalgae used in this study were* Chlorella, Scenedesmus *sp.,* Cosmarium* sp., and facultative heterotrophs (centric and pinnate diatoms) along with few photoautotrophs (*Cyclotella* and* Oedogonium*) and obligate photoautotrophs (*P. boryanum*). Effect of nutrient supplementation and the results showed that in growth phase (GP) operation with maximum N + P condition higher biomass (1.69 mg/mL) was observed, while higher lipid productivity was observed in starvation phase with maximum in C condition (28.2%) showing good wastewater treatment efficiency in terms of substrate degradation and nutrient removal during the growth phase operation. Moreover, when supplemented with CO_2_ sparging period and interval, it influences growth and lipid accumulation of microalgae cultivated in domestic wastewater under mixotrophic microenvironment. Sparging period of 120 s documented maximum biomass growth (GP, 3.4 mg/mL) and lipid productivity (SP, 27.3%), while, in intervals, 4 h (120 s) condition showed maximum biomass (3.2 mg/mL) and lipid productivity (27.8%). Fatty acid composition revealed high degree of saturated fatty acids (SFAs) varied with the experimental variations signifying their utility as biodiesel [91]. They also documented microalgal efficiency to utilize acid-rich effluents from biohydrogen production process as carbon source for lipid accumulation under heterotrophic nutritional mode. Two types of substrates, namely, synthetic volatile fatty acids (SVFAs) and fermentative fatty acids (FFAs) collected from acidogenic H_2_ producing bioreactor were used for evaluating the lipid accumulation potential in microalgae. Comparatively, FFAs documented higher biomass and lipid productivity (1.42 mg/mL (wet weight); 26.4%) than SVFAs 0.60 mg/mL; 23.1%) [92].

In another study, fatty acid methyl ester (FAME) analysis of* A. protothecoides* UMN280 showed that the microalgal lipids were mainly composed of C_16_/C_18_ fatty acids (accounting for over 94% of total fatty acid) making it suitable for high-quality biodiesel production [36]. He et al. [93] checked the feasibility of cultivating* Chlorella vulgaris* with wastewater containing high ammonia nitrogen concentrations. This study found that increasing NH_4_^+^-N from 17 to 207 mg L^−1^ yielded additional short-chain and saturated fatty acids. Lipid productivity peaked in its value of 23.3 mg L^−1^ d^−1^ at 39 mgL^−1^  NH_4_^+^-N. Hence, microalgae components could be manipulated by NH_4_^+^-N concentration of the initial feeds. The biomass and lipid productvities of some of the microalgal species grown in different wastewater resources reported till date is given in Table 1.

## 7. Nutrient Removal Efficiency

Growing algae depends on the availability of principal nutrients like nitrogen, phosphorus, carbon, sulphur and micronutrients including silica, calcium, magnesium, potassium, iron, manganese, sulphur, zinc, copper, and cobalt. Algal cells have the capability to uptake nitrogen and phosphorus from water [94, 95]. Microalgae can be efficiently used to remove significant amount of nutrients because they need high amounts of nitrogen and phosphorus for protein (45–60% of microalgae dry weight), nucleic acid, and phospholipid synthesis [12]. The nitrogen in sewage effluent arises primarily from metabolic interconversions of extra derived compounds, whereas 50% or more of phosphorus arises from synthetic detergents [27]. Nitrogen and phosphorus are the two important nutrient compounds to analyze a water source for potential algae growth. The principal forms in which they arise in wastewater are NH_4_ (ammonia), NO^−2^ (nitrite), NO^−3^ (nitrate), and PO_4_^3−^ (orthophosphate). The nutrient removal efficiency of some of the microalgal species reported till date is given in Table 2.

Algal growth and nutrient removal characteristics of microalgae* Chlorella vulgaris* using artificial wastewater in batch experiments showed that* C. vulgaris *can completely remove up to 21.2 mg L^−1^ ammonia-nitrogen concentration but showed low phosphorus removal with 7.7 mg L^−1^ initial PO_4_-P concentration with 78% efficiency [94]. A promising strain* A. protothecoides* UMN280 isolated from a local municipal wastewater plant shows high nutrient removal efficiency as well as its high growth rate and lipid productivity. The results of the six-day batch cultivation showed that the maximal removal efficiencies for total nitrogen, total phosphorus, chemical oxygen demand (COD), and total organic carbon (TOC) were over 59%, 81%, 88%, and 96%, respectively, with high growth rate (0.490 d^−1^), high biomass productivity (269 mg L^−1^ d^−1^), and high lipid productivity (78 mg L^−1^ d^−1^) [36]. Studies have demonstrated ability of* Euglena sp*. originally isolated from the sewage treatment plants and showing good lipid content of 24.6% (w/w), efficient nutrient uptake within a short span of eight days, and profuse biomass productivity (132 mg L^−1^ d^−1^) [44].

Sturm and Lamer [96] do an assessment on energy balance of microalgal production in open ponds coupled with nutrient removal from wastewater energy for algal biodiesel production. They studied microalgal yields and nutrient removal rate from four pilot scale reactors (2500 gallons each) fed with wastewater effluent from a municipal wastewater treatment plant for six months, using a total of 12 million gallons per day processed by the wastewater treatment plant. Hence, it shows that the direct combustion of algal biomass may be a more viable energy source than biofuel production, especially when the lipid content of dry biomass (10% in this field experiment) is lower than the high values reported in lab scale reactors (50 60%). Yang et al. [97] examined nutrients usages to generate 1 kg of microalgae biodiesel using nonrecycled freshwater. It will require 3726 kg of water, 0.33 kg of nitrogen, and 0.71 kg of phosphate and also shows decrease of water and nutrients usage by 84% and 55% using recycling harvest water and reduction in 90% of water requirement, eliminating the need for all the nutrients except phosphate by using sea/wastewater as culture medium. In another study, 1L biodiesel was produced consuming nutrient between 0.23 and 1.55 kg nitrogen and 29–145 g of phosphorus depending on the cultivation conditions for microalgae [98].

## 8. Conclusion

Presently, key bottleneck of biofuel production from microalgae is that the current technologies do not allow an economic and sustainable biofuel production at today's energy prices, although high biomass, lipid productivity, and nutrient removal efficiency of wastewater grown microalgae make them promising as a feedstock for renewable energy. Furthermore, there is need to analyse nutrient consumption rates of wastewater derived algal biofuels, bioprospecting of different wastewater habitats to explore indigenous oil producing microalgal strains. Moreover, efforts should be made by focusing research on development of large-scale cost-effective cultivation systems. The coupling of microalgae cultivation with wastewater might provide possibility of phycoremediation, CO_2_ sequestration, and low cost nutrient supply for the algal biomass utilization which will enhance the economic outlook of microalgae-based biofuel production systems.

## Figures and Tables

**Table 1:** Biomass and lipid productivities of microalgae grown in different wastewater resources.

Microalgae species	Wastewater type	Biomass productivity (mg L^−1^ d^−1^)	Lipid content (% DW)	Lipid productivity (mg L^−1^ d^−1^)	Reference
*Chlorella pyrenoidosa *	Activated sludge extract	11.55	NA	NA	[28]
*Chlorella pyrenoidosa *	Digested sludge Extract	51.82	NA	NA	[28]
*Chlorella pyrenoidosa *	Settled sewage	275	NA	NA	[29, 30]
*Chlorella pyrenoidosa *and* Scenedesmus *sp.	Activated sewage	92.31	NA	NA	[29, 30]
*Botryococcus braunii *	Secondarily treated sewage	35.00	NA	NA	[31]
*Scenedesmus *sp.	Artificial Wastewater	126.54	12.80	16.2	[32]
Polyculture* *of* Chlorella *sp., *Micractinium *sp., *Actinastrum *sp.	Dairy Wastewater	NA	29.00	17	[33]
Polyculture of C*hlorella *sp.,* Micractinium* sp. *Actinastrum *sp.	Primary clarifier Effluent	NA	9.00	24.4	[33]
*Chlorella asccharophila *	Carpet mill	23	18.10	4.2	[34]
*Scenedesmus *sp.	Carpet mill	126.54	12.80	16.2	[34]
*Chlorella *sp.	Centrate Muinicipal Wastewater	231.4	33.53	77.5	[35]
*Hindakia *sp.	Centrate Muinicipal Wastewater	275.0	28.30	77.8	[35]
*Chlorella *sp.	Centrate Muinicipal Wastewater	241.7	30.91	74.7	[35]
*Scenedesmus *sp.	Centrate Muinicipal Wastewater	247.5	30.09	74.5	[35]
*Auxenochlorella protothecoides *	Concentrated Muinicipal Wastewater	268.8	28.9	77.7	[36]
*Chlamydomonas Mexicana *	Piggery Wastewater	NA	33 ± 3.4^a^	0.31 ± 0.03^a^	[37]
*Scenedesmus obliquus *	Piggery Wastewater	NA	31 ± 0.8^a^	0.24 ± 0.03^a^	[37]

*NA: Not Available.

^a^Expressed in dwt/L.

**Table 2:** Nutrient removal efficiency of microalgal species.

Microalgal species	Wastewater type	Nitrogen	Phosphate	COD	Reference
*Chlorella vulgaris *	Textile wastewater	(44.4–45.1%)	(33.1–33.3%)	(38.3–62.3%)	[38]
*Scenedesmus *sp. LX1	Modified effluent of a wastewater treatment plant of an electric factory by photo-membrane bioreactor	46%	100%	NA	[39]
*Chlorella sorokiniana* and aerobic bacteria	Potato processing industry	>95	80.7	84.8	[40]
*Chlorella sorokiniana* and aerobic bacteria	Pig manure	82.7	58.0	62.3	[40]
*Chlamydomonas* sp. TAI-2	Industrial wastewater	100%	33%	NA	[41]
*Auxenochlorella protothecoides* UMN280	Concentrated municipal wastewater	59%	81%	88%	[36]
*Chlorella Mexicana *	Piggery wastewater	62%	28%	NA	[37]
*Scenedesmus obliquus *	Piggery effluent	23–58%	48–69%	NA	[42]
*Chlamydomonas Polypyrenoideum *	Dairy industry wastewater	74%–90%	70%	NA	[43]
*Euglena* sp.	Sewage treatment plant	93%	66%	NA	[44]

*NA: Not Available.
